# The relationship between inequitable gender norms and provider attitudes and quality of care in maternal health services in Rwanda: a mixed methods study

**DOI:** 10.1186/s12884-021-03592-0

**Published:** 2021-02-22

**Authors:** Kate Doyle, Shamsi Kazimbaya, Ruti Levtov, Joya Banerjee, Myra Betron, Reena Sethi, Marie Rose Kayirangwa, Kristina Vlahovicova, Felix Sayinzoga, Rosemary Morgan

**Affiliations:** 1 Promundo-US, 1367 Connecticut Avenue, NW, Suite 310, Washington, DC 20036 USA; 2 Present Address: The Prevention Collaborative, Washington, DC, USA; 3 Present Address: CARE, 1899 L St NW #500, Washington, DC 20036 USA; 4 Maternal and Child Survival Program/Jhpiego, 1776 Massachusetts Ave, NW, Suite 300, Washington, DC 20036 USA; 5 Maternal and Child Survival Program/Jhpiego, 8 Avenue, Rwanda National Police (RNP Road), Kigali, Rwanda; 6 Maternal, Child and Community Health Division, Rwanda Ministry of Health, Rwanda Biomedical Center, Kigali, Rwanda; 7 Department of International Health, Johns Hopkins Bloomberg School of Public Health, 615 N Wolfe St, Baltimore, MD 21205 USA

**Keywords:** Gender, Quality of care, Labor and childbirth, Mistreatment, Disrespect and abuse, Antenatal care, Maternal and newborn health, Rwanda, Mixed methods

## Abstract

**Background:**

Rwanda has made great progress in improving reproductive, maternal, and newborn health (RMNH) care; however, barriers to ensuring timely and full RMNH service utilization persist, including women’s limited decision-making power and poor-quality care. This study sought to better understand whether and how gender and power dynamics between providers and clients affect their perceptions and experiences of quality care during antenatal care, labor and childbirth.

**Methods:**

This mixed methods study included a self-administered survey with 151 RMNH providers with questions on attitudes about gender roles, RMNH care, provider-client relations, labor and childbirth, which took place between January to February 2018. Two separate factor analyses were conducted on provider responses to create a Gender Attitudes Scale and an RMNH Quality of Care Scale. Three focus group discussions (FGDs) conducted in February 2019 with RMNH providers, female and male clients, explored attitudes about gender norms, provision and quality of RMNH care, provider-client interactions and power dynamics, and men’s involvement. Data were analyzed thematically.

**Results:**

Inequitable gender norms and attitudes – among both RMNH care providers and clients – impact the quality of RMNH care. The qualitative results illustrate how gender norms and attitudes influence the provision of care and provider-client interactions, in addition to the impact of men’s involvement on the quality of care. Complementing this finding, the survey found a relationship between health providers’ gender attitudes and their attitudes towards quality RMNH care: gender equitable attitudes were associated with greater support for respectful, quality RMNH care.

**Conclusions:**

Our findings suggest that gender attitudes and power dynamics between providers and their clients, and between female clients and their partners, can negatively impact the utilization and provision of quality RMNH care. There is a need for capacity building efforts to challenge health providers’ inequitable gender attitudes and practices and equip them to be aware of gender and power dynamics between themselves and their clients. These efforts can be made alongside community interventions to transform harmful gender norms, including those that increase women’s agency and autonomy over their bodies and their health care, promote uptake of health services, and improve couple power dynamics.

**Supplementary Information:**

The online version contains supplementary material available at 10.1186/s12884-021-03592-0.

## Background

In recent years, Rwanda has made great progress in improving reproductive, maternal, and newborn health (RMNH) care by investing in health workforce development, scaling up service provision, and increasing the demand for, accessibility and quality of health services. According to Rwanda’s 2014/15 Demographic and Health Survey (DHS), the maternal mortality ratio was more than halved between 2010 and 2015, falling from 476 per 100,000 live births to 210 [1]. By 2015, nearly all women attended at least one antenatal care (ANC) visit (99.0%) and gave birth in a health facility (90.7%) [1]. The country has also adopted strategies to promote men’s engagement in RMNH, including participation in ANC and family planning services, in order to address barriers to care and to improve service uptake [2, 3].

Despite many advances, challenges to ensuring timely and full RMNH service utilization remain. The DHS indicated that only 44% of women attended the recommended four ANC visits – which has since been increased by the World Health Organization (WHO) to a recommended eight visits – and only 56% of women sought ANC before their fourth month of pregnancy [1]. Women in the lowest socio-economic level and with the least amount of education were the least likely to deliver in a health facility [1]. In addition, 55% of women reported that they did not have a postnatal check-up [1]. The DHS noted that barriers to women’s care seeking or to timely care were varied and multiple, but included cost, the distance to the health facility, and women’s limited decision-making power [1].

Recent research in Rwanda has found that women’s dissatisfaction with and experiences of low-quality care during pregnancy were disincentives for attending the recommended number of ANC visits and were catalysts for seeking care at another health facility [4]. Women reported mistrust of health providers and being dissatisfied with the care they received for varied reasons, including receiving limited or superficial information, or not being able to discuss their personal concerns [4]. As a result, some women sought out traditional healers, while others reported that the inadequate care they received in facilities led them to seek care for the same, unresolved problem multiple times. The researchers also found that men believed their presence at ANC or delivery was important for ensuring their partners received better quality care [5].

The WHO defines quality of care as “the extent to which health care services provided to individuals and patient populations improve desired health outcomes. In order to achieve this, health care must be safe, effective, timely, efficient, equitable and people-centred”; which includes ensuring individuals receive the same quality of care regardless of gender and that care takes into account individual preferences [6]. Recent research has highlighted the important role that quality, respectful RMNH care has for women’s care-seeking and positive birth experiences and outcomes. Studies in multiple settings have found that a lack of privacy, uncaring provider attitudes, physical abuse, and delays in receiving care led to dissatisfaction with the care women received, and were deterrents from giving birth in a health facility or seeking facility based-care for complications [7–12]. In many low-income settings, fear of mistreatment and abuse during childbirth has been found to be a reason why women avoid giving birth in a facility [13, 14]. Evidence shows that globally many women experience mistreatment and abuse during childbirth, which includes abusive, neglectful or disrespectful care [13]. Disrespect and abuse during childbirth has been defined as “interactions or facility conditions that local consensus deems to be humiliating or undignified, and those interactions or conditions that are experienced as or intended to be humiliating or undignified” [15]. Bowser and Hill identified seven categories of disrespect and abuse in maternity care, including “physical abuse, non-consented clinical care, non- confidential care, non-dignified care (including verbal abuse), discrimination based on specific patient attributes, abandonment of care, and detention in facilities” [16].

Research from other low-and-middle-income settings in Asia and Africa suggests that health providers’ attitudes about gender roles may influence their interactions with female and male clients at RMNH services, and ultimately impact the quality of care (QOC) female clients receive [17]. Studies examining the impact of men’s participation at ANC or family planning services have found that men’s presence may sometimes negatively impact providers’ interactions with female clients. For example, some health providers have been reported to pay greater attention to men than to women in these situations [18], or to be more likely to respond supportively to men’s comments and questions and to disagree with or ignore women’s input [19]. Studies examining maternal health providers’ interactions with clients have also found that QOC was influenced by providers’ beliefs about appropriate roles and behavior and prejudices towards certain client attributes, such as their socio-economic status, age or education level [20].

While existing studies in Rwanda have examined providers’ and clients’ experiences and perspectives of male involvement in RMNH services, they have not adequately explored how or if health providers’ gender attitudes and perceptions of female and male clients influence client-provider interactions. This study sought to build on existing studies and contribute to addressing gaps in the research on RMNH care in Rwanda, to better understand whether and how gender and power dynamics between providers and clients affect their perceptions and experiences of the quality of RMNH care. This paper examines several of the study’s objectives, to understand: health providers’ attitudes towards gender roles and norms, and their perceptions of male and female clients attending RMNH services; health providers’ experience of RMNH service provision and quality of care; and women’s and men’s experiences and perceptions of accessing RMNH services and quality of care. The paper presents findings related to maternal health services, including ANC, labor and childbirth.

## Methods

This mixed methods study was conducted in Rwanda by the USAID Maternal and Child Survival Program (MCSP), through Promundo-US, and in collaboration with the Rwanda Ministry of Health and the Rwanda Biomedical Center. Ethical approval was obtained from the Rwanda National Health Research Committee (NHRC/2017/PROT/036), the Rwanda National Ethics Committee (No. 633/RNEC/2017), and the Johns Hopkins School of Public Health Internal Review Board (IRB00008319). Informed consent was obtained from all study participants.

The study was conducted in 31 public health facilities including referral/district hospitals, health centers and their catchment areas in three districts (out of 30): Kamonyi (Southern Province); Ngoma (Eastern Province); and Nyabihu (Western Province). The survey was administered in all three districts, while qualitative data collection was limited to Ngoma district due to funding constraints. The districts were selected from among the 10 districts where MCSP, an integrated reproductive, maternal and newborn health program, was active. At the time of the study, MCSP was supporting the implementation of a community-based. Gender-transformative, male engagement intervention in six of the 10 districts. In order to avoid biasing findings, the three districts were chosen from among the four where MCSP was not implementing this intervention. The three districts selected were chosen to represent both rural and peri-urban settings and all three provinces where MCSP was implementing interventions.

The proportion of the population living in poverty in the selected districts is lower than the national average (38.2%) in Kamonyi (22.3%) and Ngoma (37.8%), but higher in Nyabihu (46.8%) [21]. All three districts have high rates of antenatal care utilization (at least once) during pregnancy (from 98.1 to 100.0%) and facility-based delivery (from 88.4 to 92.4%), although the rates in Ngoma district are below the national average [1]. A considerable proportion of women in all three districts report at least one problem when accessing health care for themselves, with rates in both Ngoma (69.4%) and Nyabihu (64.3%) above the national average (58.6%) [1]. The challenge most cited by women in all three districts was getting money for treatment, followed by distance to the health facility, not wanting to go alone, and getting permission to go for treatment [1]. While 97.6% of women in Nyabihu district report participating in decisions about their own health care – whether alone or jointly with a partner, fewer women in Ngoma (83.6%) and Kamonyi (79.2%) district reported participating in these decisions [1], which is likely to hinder their health care seeking during pregnancy.

### Recruitment and data collection

The study collected data from three populations: 1) RMNH providers; 2) female clients attending RMNH services, and 3) male clients attending or accompanying a partner to RMNH services. Eligibility criteria for inclusion in the study were: RMNH providers were employed as doctors, nurses or midwives in public health facilities and providing one or more RMNH services, including ANC, maternity, post-natal care, or family planning (the latter two services were included in the larger study but not reported on here). Female clients were at least 18 years old and had given birth in one of the health facilities selected for the study within the past 2 years. Male clients were at least 18 years old, and had accompanied a partner (who had given birth in the past 2 years) to an RMNH service at a health facility selected for the study within the past 2 years. RMNH providers were eligible for both quantitative and qualitative data collection; clients were included in qualitative data collection only.

#### Quantitative sample

Recruitment and data collection with RMNH providers took place from January to February 2018. We determined a minimum required sample size of 146 health providers from across all three districts (49 per district) using a conservative value estimate of *p* = 50%, desired precision of +/− 10, 80% power and alpha = .05, along with an adjustment for a conservative design effect of 1.5. The desired sample size was then rounded up to an even 50 providers per district (a total of 150). The sample size also met rule of thumb criteria of a minimum of 10 respondents per item for factor analysis [22]. Within each district, respondents were recruited from a total of 10 health facilities: the district/referral hospital and nine randomly selected health centers, selected from the full list of health centers in each district (excluding prison-based facilities) using a random number generator in STATA Version 14 (Stata Corporation, College Station, TX). On the day of the survey, convenience sampling was used to select eligible providers. Upon reaching each facility, the study team made separate, sex-disaggregated lists of providers who were present in the facility and met the eligibility criteria. Every other provider, alternating from each sex-disaggregated list, was then invited to participate (1 female, 1 male, and so on), until the desired sample size at facility and district level was reached. The number of providers recruited from each facility was capped at 10 respondents for hospitals and five for health centers, to ensure that providers were recruited from all 10 selected facilities per district. Equal numbers of male and female providers were sought to enable comparison between male and female providers’ responses, but the ratio of male to female providers surveyed was limited by the sex distribution of providers working in the district, and the number and sex of eligible providers present on the day the survey was administered. Ultimately 151 respondents participated (61 men and 90 women), out of a total of 295 eligible providers.

#### Qualitative sample

Recruitment and data collection for focus group discussions (FGDs) took place in February 2019. Convenience sampling was used to select respondents from a sub-sample of selected health facilities and their catchment areas in Ngoma district. The qualitative study was limited to one district due to financial and human resource constraints. Eligible RMNH providers were recruited from the district hospital and two health centers. Eligible clients were recruited from the catchment areas of two health centers. The health facilities selected were located in close proximity to each other, to minimize respondents’ travel time. The study team received lists of eligible RMNH providers (disaggregated by sex) from health facility managers, and of eligible female and male clients willing to participate in the study from local community health workers. The study team contacted every other individual on the respective lists by telephone to verify eligibility and enroll willing participants until the desired sample size was reached.

Three separate FGDs were conducted with 10 RMNH providers (five men, five women; five nurses, four midwives, one doctor), 10 female clients, and 10 male clients, respectively. The FGDs were conducted in Kinyarwanda by two Rwandan researchers (one male and one female) trained by the research team; FGDs with clients were conducted by sex-matched interviewers. The discussions were conducted in rooms with auditory privacy within public buildings such as sector offices, schools or health centers, and lasted on average one and a half to 2 h. All FGD participants were provided with a transportation stipend of 3000 Rwandan francs to enable their participation. Mothers who attended the FGD with infants were offered food/beverages.

### Data collection tools and analysis

#### Quantitative study

Quantitative data collection consisted of a 32-question survey developed for this study (Additional File 1), which examined: providers’ attitudes related to gender roles and relations, the provision and quality of RMNH care, and provider-client interactions and power dynamics related to labor and childbirth. The surveys were self-administered by recruited health providers and then placed in sealed envelopes and collected by the research team. Fourteen questions on the survey examined gender attitudes related to gender roles, intimate partner violence, household decision-making, sex, and reproduction. The questions were inspired by or adapted from the Gender Equitable Men (GEM) Scale [23] and the International Men and Gender Equality Survey (IMAGES). Fourteen questions examined attitudes related to RMNH service provision and QOC, all of which were created or adapted for the study based on relevant literature describing dimensions of QOC and disrespect and abuse [16, 24], discussions with the study team, and knowledge of the local context. For the attitude questions, respondents were asked the degree to which they agreed with particular statements (1 = strongly agree, 2 = agree, 3 = disagree, or 4 = strongly disagree). Most statements were phrased such that disagreement with them indicated a more equitable attitude, with the exception of five statements for which agreement indicated a more equitable attitude. These five statements were reverse coded (1 = strongly disagree, 2 = disagree, 3 = agree, or 4 = strongly agree), such that higher scores indicate more equitable attitudes.

When analyzing the survey data, frequencies were tabulated for the full sample, disaggregated by sex. Statistical differences between male and female providers were examined using chi-squared tests and reported whenever significance levels were *p* < .05. Following data collection, two separate factor analyses were conducted on providers’ responses to the gender attitude statements and the RMNH quality of care statements, to enable the creation of a Gender attitudes scale and an RMNH QOC attitudes scale, respectively. To create a gender attitudes scale, we conducted a factor analysis starting with 13 of the 14 gender attitude items, restricting the solution to a single factor, and retaining items with factor loadings of 0.4 and above. The solution was restricted to one factor to allow comparability to another ongoing study in Rwanda. One statement (“adolescents seeking contraceptives should be advised to abstain from sex”) was excluded from the factor analysis because it did not specifically relate to gender. The analysis included data from 141 respondents with no missing values. This resulted in a 9-item gender attitude scale with an acceptable internal reliability coefficient (Cronbach’s alpha = 0.69). A gender attitudes score was then constructed for each individual by taking the mean across the 9 items, generating a score ranging from 1 to 4, with higher scores indicating more equitable attitudes.

We also conducted an exploratory factor analysis on the responses to the 14 statements on RMNH quality of care to create an RMNH QOC attitude scale (including responses from 142 service providers with no missing values across the items). This resulted in a 12-item scale with a borderline acceptable alpha of 0.62. An RMNH QOC attitudes score was then constructed for each respondent across the 12 items, ranging from 1 to 4, with higher scores indicating attitudes more supportive of respectful or quality RMNH care. A multivariate ordinary least squares (OLS) regression model was then used to examine the associations between the scores on the two scales (gender attitudes and RMNH QOC attitudes), controlling for demographic factors such as age, sex, and type of service provided (i.e. provides both family planning (FP) and maternal or newborn health (MNH) services vs. providing only one or neither of these services). The full study explored the broader range of RMNH services, including family planning services. As such, our analysis included examining any differences in attitudes between providers who solely provided family planning services and those who also provided maternal and newborn health services.

#### Qualitative study

Vignettes – or brief, evocative case studies – of hypothetical women and men attending RMNH services were utilized in the three FGDs (with providers, female clients, male clients) to explore respondents’ perceptions and experiences of accessing or providing RMNH care. Vignettes can be a useful tool for exploring social norms and sensitive topics, such as violence or abusive care, which respondents may otherwise feel uncomfortable to discuss [25, 26]. For example, discussing fictional scenarios can be a less personal or threatening way to discuss topics than responding to direct questions about their own opinions and experiences, and can enable respondents to decide if or when to share their own experiences. The vignettes in this study were developed based on team discussions, a review of relevant and country specific research, and situations and trends encountered by MCSP during project implementation in Rwanda. They included scenarios of fictional women attending ANC, maternity and family planning services – including one describing an instance of obstetric violence – which were tailored to each study population. The scenarios touched on issues of quality of RMNH care, provider-client interactions, disrespect and abuse, and men’s presence at RMNH services, for example:

*Claudine is now pregnant for the first time. She has been married for about 18 months, and she and her husband Jean Marie have a small plot of land that they farm, which has allowed them to purchase health insurance. Let’s think about Claudine’s first visit to the antenatal clinic. Her husband joined her for this visit, as recommended.*

Respondents were asked how they imagined or believed the characters in the situations would or should act, including how provider-client interactions might differ depending on a woman’s age, number of pregnancies or whether she was accompanied by a partner. Semi-structured discussion questions were also used to further explore themes of provider-client interactions, barriers to and quality of RMNH care, and men’s attendance at RMNH services.

The audio-recordings of the FGDs were translated and transcribed verbatim from Kinyarwanda into English by a professional translator. Three of the authors (SK, FS, MK) listened to the audio files and verified translation and transcription, after which the audio recordings were deleted to ensure confidentiality of respondents. The analysis included both deductive and inductive approaches. A coding structure was developed based on the FGD guides and existing research and further refined based on the data in the transcripts, to enable thematic analysis [27]. Transcripts were coded and analyzed in Dedoose software by a member of the research team and reviewed by the lead author. Emerging findings were discussed by the lead author with members of the research team to verify the analysis and interpretation of the data.

## Results

Table 1 describes the demographics of the 151 RMNH providers (90 women, 61 men) who completed the survey. The proportion of male providers in our sample (40.4%) is somewhat greater than that of the RMNH care providers serving in the health facilities within the selected districts (33.0%) at the time of the study, based on information obtained from the heads of those health facilities. The overwhelming majority (95%) of health providers responding to the survey were midwives or nurses, while 4% were doctors and 1 person identified their occupation as “other.” This distribution reflects the general distribution of RMNH care providers serving in health facilities within the selected districts at the time of the study (5% doctors; 79% nurses; 16% midwives). Almost two-thirds of the respondents (64%) reported providing both family planning and at least one MNH service, such as ANC, labor/delivery services, or postnatal care.

**Table 1:** Health provider demographics disaggregated by sex

	Men		Women		TOTAL	
	%	n	%	n	%	n
**Sex distribution of sample**	40.4	61	59.6	90	100.0	151
	Mean	SD	Mean	SD	Mean	SD
**Age**	34.03	6.19	35.77	6.55	35.07	6.44
**Age groups**	%	n	%	n	%	n
Younger (< 35)	57.38	35	47.78	43	51.66	78
Older (35+)	42.62	26	52.22	47	48.34	73
**Occupation**	%	n	%	n	%	n
Nurse or midwife	91.80	56	97.78	88	95.36	144
*Midwife (A1)*	6.56	4	18.89	17	13.91	21
*Nurse (A1)*	45.90	28	38.89	35	41.72	63
*Nurse (A2)*	39.34	24	38.89	35	39.07	59
*Nurse (A3)*	0.00	0	1.11	1	0.66	1
Doctor	8.20	5	1.11	1	3.97	6
*Doctor (generalist)*	4.92	3	1.11	1	2.65	4
*Doctor (specialist)*	3.28	2	0	0	1.32	2
Other	0.00	0	1.11	1	0.66	1
**% of respondents who provide the following services**	%	n	%	n	%	n
Family planning	65.57	40	70.00	63	68.21	103
Antenatal care	60.66	37	76.67	69	70.20	106
Labor/delivery	81.97	50	78.89	71	80.13	121
Postnatal care	77.05	47	77.78	70	77.48	117
Other service	49.18	30	40.00	36	43.71	66
**% of respondents who provide both FP and MNH (antenatal, labor/delivery, postnatal) services**	62.30	38	65.56	59	64.24	97

*A1 midwives have completed 3 years of training in midwifery at an institution of higher education; A1 nurses have completed 3 years training in nursing at an institution of higher education (A1 nurses are registered nurses); A2 nurses have completed 6 years of secondary school training with a general nursing focus; A3 nurses have 3 years of post-primary school training in nursing*

### Health providers’ gender attitudes

Table 2 describes the findings on RMNH providers’ responses to all 14 gender attitude statements included in the survey. Overall, the gender attitudes score of this sample of providers was 3.28 (SD = 0.35) on a scale of 1 to 4, with higher scores indicating more equitable gender attitudes. The gender attitudes scale score was standardized (mean = 0, SD = 1) in the regression analysis for ease of interpretation. The items included in the gender attitudes scale are denoted with a number sign (#) in Table 2. There was no significant difference between the gender attitudes scores of male and female providers.

**Table 2:** Health providers’ gender attitudes disaggregated by sex

	Male Providers		Female Providers		TOTAL		***p***-value for difference between male and female providers
	Agree or strongly agree		Agree or strongly agree		Agree or strongly agree		
	%	***n*** = 61	%	***n*** = 90	%	n	
1. Men should be as involved in caring for their children as women are. #&	100.0	59	100.0	90	100.0	149	*p* = 0.654
2. Women should remain virgins until they get married.	81.4	48	88.9	80	85.9	128	*p* = 0.496
3. A woman should be able to use contraceptives, even if her husband disagrees.	70.0	42	79.77	71.0	75.8	113	*p* = 0.262
4. Adolescents seeking contraceptives should be advised to abstain from sex.	76.7	46	70.0	63	72.7	109	*p* = 0.531
5. A woman’s most important role is to take care of her home and cook for her family.	57.6	34	74.2	66	67.6	100	*p* = 0.112
6. A man should have the final word about decisions in his home. #	28.8	17	23.6	21	25.7	38	*p* = 0.888
7. It is a woman’s responsibility to avoid getting pregnant. #	17.2	10	28.1	25	23.8	35	*p* = 0.430
8. A woman should not use a family planning method unless her partner agrees.	30.5	18	18.0	16	23.0	34	*p* = 0.244
9. It is natural and right that men should have more power than women in the family. #	22.0	13	22.5	20	22.3	33	*p* = 0.993
10. Men are better at making decisions than women are. #	13.6	8	13.6	12	13.6	20	*p* = 0.974
11. There are times when a woman deserves to be beaten. #	3.4	2	0.0	0	1.3	2	*p* = 0.370
12. A woman who uses contraceptives without telling her husband deserves to be beaten. #	0.0	0	1.1	1	0.7	1	*p* = 0.300
13. A woman who has not undergone *gukuna imishino* (labia elongation) does not deserve respect from her husband. #	0.0	0	1.1	1	0.7	1	*p* = 0.110
14. A girl who is pregnant before marriage deserves to be shunned, sent away, or otherwise punished. #	1.7	1	0.0	0	0.7	1	*p* = 0.470

*Note: % agree reported out of all responses to each particular item; missing responses ranged from 1 to 4 out of 151. Chi-squared tests were used to assess differences in full response distributions between male and female providers*

*#: item included in the ‘gender attitudes scale’*

*&: item was reverse coded for analysis*

All RMNH providers surveyed agreed or strongly agreed that men should be as involved in caring for their children as women are. However, there was less consensus in responses to other statements related to men’s and women’s roles within the family. For example, 25.7% of providers agreed that a man should have the final word about decisions in his home and 22.3% agreed that it is natural and right for men to have more power than women in the family. Across all gender attitude items, there were no significant differences by sex of provider.

### Health providers’ attitudes about RMNH QOC

Table 3 describes the findings on RMNH providers’ responses to all 14 attitude statements related to the provision and quality of RMNH care, including provider-client interactions and power dynamics. The overall RMNH QOC attitude score for this sample of providers was 3.07 (SD = 0.34) on a scale of 1 to 4, with higher scores indicating attitudes more supportive of respectful or quality RMNH care. The 12 statements included in the RMNH QOC attitude scale constructed for this study are denoted by a number sign (#) in Table 3. There was no significant difference between the scale scores of male and female providers.

**Table 3:** Health providers’ attitudes about RMNH care and quality disaggregated by sex

	Male Providers		Female Providers		TOTAL		***p***-value for difference between male and female providers
	Agree or strongly agree		Agree or strongly agree		Agree or strongly agree		
	%	*n* = 61	%	*n* = 90	%	n	
1. When health providers listen to their clients’/patients’ questions and concerns, it results in better service provision. #&	100.0	59	100.0	88	100.0	147	*p* = 0.910
2. Health providers should show sympathy and care to women during childbirth. #&	100.0	60	100.0	90	100.0	150	*p* = 0.779
3. A woman should be allowed to have a companion present at delivery, if she chooses, and without any further explanation. #&	98.3	58	96.6	86	97.3	144	*p* = 0.822
4. It is easier to work with women when they come to ANC with their partners.	95.0	57	97.8	88	96.7	145	*p* = 0.342
5. A woman should be able to get help from a skilled birth attendant when she needs it, even without her husband’s permission. #&	88.1	52	90.8	79	89.7	131	*p* = 0.523
6. Sometimes you have to yell or shout at a woman while she is giving birth to get her to push. #	37.3	22	24.7	22	29.7	44	*p* = 0.191
7. A good client never questions a health provider’s decisions, even if s/he disagrees with them. #	21.7	13	27.8	25	25.3	38	*p* = 0.495
8. It is important for health providers to assert their power over their clients in order to be respected. #	25.4	15	21.8	19	23.3	34	*p* = 0.940
9. A good woman keeps quiet during delivery, even when she is in pain. #	16.9	10	24.7	22	21.6	32	*p* = 0.170
10. It is better for a health provider to be decisive than to explain everything to a client/patient. #	13.3	8	18.0	16	16.1	24	*p* = 0.681
11. Male health providers have more power and are more respected than female health providers.	16.7	10	4.4	4	9.3	14	*p* = 0.006
12. Men should not be present when women are giving birth. #	6.8	4	9.1	8	8.2	12	*p* = 0.050
13. If a woman is unable to pay for the services she has received, she should be detained in the facility until payment is made. #	8.5	5	3.4	3	5.4	8	*p* = 0.292
14. Sometimes women need a little push or slap to motivate them during childbirth. #	3.4	2	4.5	4	4.0	6	*p* = 0.561

*Note: % agree reported out of all responses to each particular item; missing responses ranged from 1 to 5 out of 151. Chi-squared tests were used to assess differences in full response distributions between male and female providers*

*#: item included in the ‘RMNH QOC scale’*

*&: item was reverse coded for analysis*

All providers agreed that health providers should show sympathy and care to women during childbirth. However, a notable proportion of providers demonstrated acceptance of disrespectful or abusive attitudes and treatment. For example, nearly 30.0% of providers agreed that sometimes you have to yell or shout at a woman while she is giving birth to get her to push and 10.6% did not agree that a woman should be able to get help from a skilled birth attendant when she needs it without her husband’s permission.

A multivariate OLS regression model was used to examine the associations between the scores on the two scales (gender attitudes and RMNH QOC attitudes), controlling for demographic factors such as age, sex, and type of service provided. Since the RMNH QOC scale score distribution was skewed and regression diagnostics indicated a need for variable transformation, we used a cubic transformation for the variable in the regression. The analysis, presented in Table 4, shows a statistically significant positive association between RMNH providers’ attitudes related to the provision of quality, respectful RMNH care (using the RMNH QOC attitude scale) and their equitable gender attitudes. That is, the findings showed that providers who hold more gender equitable attitudes tend to hold more sensitive and responsive attitudes towards respectful RMNH care.

**Table 4:** Results from multivariate regression analysis of RMNH QOC

	Unadjusted			Adjusted		
Dependent variable: RMNH QOC (cubed)	Beta	SE	95% CI	Beta	SE	95% CI
Gender attitude score (standardized)	3.03***	0.71	[1.62, 4.43]	2.91***	0.72	[1.49, 4.33]
Male				−0.257	1.46	[−3.15, 2.63]
Age				−0.098	0.11	[−0.32, 0.12]
Provides both Family Planning and MNH services (ref = does not provide both FP and MNH services)				1.98	1.48	[−0.94, 4.91]
***R-squared***			0.11			0.12

Notes: *** p < 0.001, ** p < 0.01, * p < 0.05, CI=Confidence interval

### Qualitative findings on perceptions and experiences of quality of care

#### Provider-client interactions and the quality of RMNH care

In the qualitative research, female and male clients described positive provider-client interactions as a defining aspect of quality RMNH care. Both female and male clients emphasized how the initial interaction with a provider shaped their perceptions of the service quality, and described the need for a friendly reception by the health provider. Several female clients described the importance of providers being kind, spending time, and listening attentively to women attending services, which they noted were important for developing trust and alleviating their anxieties. One female client suggested the health provider’s behavior determines how comfortable a woman is; another noted that when trust is not built, women may withhold vital information from the provider, which can undermine their care. Female clients also emphasized the importance of providers explaining the recommendations and care being given.

When discussing poor quality RMNH care, female and male clients described providers using harsh, unkind or “bad” language. Women and men both noted that clients can be afraid of health providers and suggested a fear of being spoken to badly can deter people from attending RMNH services. Male clients in particular complained about health providers who were distracted or delayed providing care, for example by being on the phone (for personal reasons). One male client stated that it can make a person feel *“that there is something more important to the health provider.”* Clients also described how negative experiences or interactions with providers can deter future care-seeking at the same facility or with a particular provider, for themselves, as well as their partners and friends. However, several clients suggested that most health providers treat people kindly these days, and negative interactions are due to a provider’s personality, as shared by this female client;

*“Sometimes a health care provider behaves badly because that is her/his personality, and when you ask her/him something she/he responds to you in a bad way, but you don’t see many people like that. Now many are really trying … It is now rare to see a health provider who insults someone.” (female client)*

Health providers similarly described the importance of positive, kind, and friendly interactions with women and men attending RMNH services as a critical component of quality care. Several providers emphasized the need for a “warm” welcome, to make clients comfortable and reduce their fears or worries. When discussing female clients, providers emphasized the importance of developing trust, by listening and providing explanations, as highlighted by this provider: “*When they have understood well the explanations, in their hearts they start taking decisions based on what you explained*.” Providers also acknowledged that a negative interaction with a provider or receiving poor-quality care could deter a client from future care-seeking. RMNH providers described how women’s personal experiences of RMNH services in particular informed their own future care-seeking as well as that of others, as shared by one provider: “*If … we beat her, we disrespect her, she will not come back … even those who want to come, she discourages them.”*

#### Perceptions of male engagement in antenatal care

On the whole, both female and male clients showed support for men’s engagement in RMNH services, and described the added value of men’s participation, particularly at ANC. Female and male clients described men’s presence at the first ANC visit (which is strongly encouraged in Rwanda) as an opportunity for men to learn how to support a healthy pregnancy, and for couples to jointly prepare for the birth and decide on post-partum contraceptive use. Female clients described the importance of men learning this information directly from the health provider, rather than from the woman herself, as they felt men were more likely to trust and therefore follow the recommendations given. Some female clients also suggested that health providers treat women attending ANC with a partner with greater respect than women attending alone.

RMNH providers were also supportive of men’s participation in ANC and emphasized multiple benefits of men’s presence at the first ANC visit, including “teaching” men about a healthy pregnancy, HIV testing and advising on post-partum contraception. Several providers suggested that receiving the couple together made their job easier and increased the likelihood of post-partum contraceptive use by enabling consensus between the partners. One provider shared that when the couple is together, “the teachings she will give them will be understood easier than when the woman had come alone.” However, a few providers noted that women may feel less comfortable, or able to ask questions or discuss sensitive issues when their partner is present. When asked whether women attending alone received the same quality of care, most providers stated that women attending ANC with or without a partner receive the same QOC, as one provider described: *“the man’s presence or absence should not make the quality of service different”.*

However, several providers admitted to using unsanctioned practices to encourage men’s antenatal participation – such as seeing couples faster than single/unaccompanied women seeking ANC, being overly kind to male partners, or publicly praising men for their participation. These practices were informed by providers’ beliefs that men needed to be seen faster because they are impatient, do not like to spend long waiting, or have other responsibilities they need to attend to. They also described these tactics as encouraging men’s (and their peers’) future ANC participation. In addition to these practices, one provider suggested that there may be bias against women attending the first ANC visit without a partner, which could undermine the quality of care:

*“Sometimes you may not give her the services the same way you gave it to others because you were in a good mood with those couples, interacting with both the husband and the wife. So, you might give her the quality of service which is not as good as the one you gave others. We should admit that it can happen.” (RMNH provider)*

#### Expectations, care and empathy during labor and childbirth

When discussing childbirth, female clients appreciated providers who spoke kindly to them, made time for them and guided them throughout labor and childbirth. They noted the importance of providers explaining the care being given and emphasized that women need more interaction with providers during this time. Several female clients suggested that first-time mothers in particular required more attention, care and guidance; while multiparous women had a better understanding of what to expect, and therefore required less attention. A number of female clients noted the need for providers to show more respect and empathy to women during childbirth.

Most providers expressed the need to care for, advise, and have empathy for women during labor and childbirth. As one provider noted, “*pain is a personal experience and what we do is encourage her … there is nothing you can do apart from encouraging her and showing her that you are around for her*.” Several providers suggested that providers should show more respect and empathy towards women during childbirth. Similar to female clients, several providers suggested that first-time mothers require more attention, care and guidance; while multiparous women require less attention because they have a better understanding of what to expect and how to behave. However, interactions between with women during labor and childbirth were influenced by providers expectations and judgments of what was considered acceptable or appropriate behavior for women. Providers appreciated women who were quiet, submissive and obedient.

#### Perceptions and experiences of disrespectful or abusive treatment during labor and childbirth

Female clients noted that verbal and physical abuse – such as slapping or hitting a woman during labor or childbirth – happened less frequently than in the past, when it used to be common. However, they acknowledged that it sometimes still occurs, particularly if a woman is seen to be misbehaving. Several female clients suggested that verbal or physical abuse from a provider was most likely to occur when the baby is crowning and a woman closes her legs instead of pushing. One female client described her own experience of how the health provider “*shouts at you with an angry face so that you can push the baby*”. Several female clients suggested that providers may slap or hit a woman to correct her misbehavior, particularly if it threatens the life of the baby:

*“Sometimes she can behave badly and the health provider realizes that it can make the baby’s life in danger, so the health provider can slap her with the intention to bring her back to the right track in order to save the baby’s life.” (female client)*

Several female clients suggested that women will forget about the abuse once they see their healthy baby. They also reported that providers sometimes apologize to a woman if they spoke harshly or slapped her during the delivery. One female client suggested that a woman will actually thank the provider *“instead of accusing him/her of anything*”. However, another female client stated that while an apology from the provider *“reduces your trauma”,* the experience is not easily forgotten.

Most providers also agreed that verbal and physical abuse during labor and childbirth were less common than in the past when some described it as “normal”. However, they also acknowledged that disrespectful or abusive interactions may still occur. Most providers suggested that experiences of verbal or physical abuse were linked to women’s perceived misbehavior – such as screaming loudly or not pushing at the right moment. Providers suggested that providers may become frustrated and react unkindly to women who are perceived as misbehaving, particularly if the woman’s behavior is seen as a threat to the child’s life. Most providers agreed that such abuse was not acceptable, but felt that when it was done, it was often done out of good intentions. As described by one provider:

*“Most of the times it is done depending on the way a woman behaved. A health provider doesn’t beat her out of unkindness, she/he beats her to save the baby. Sometimes a health provider does it when he/she sees that if she does another step, it will be over for the baby! It doesn’t happen often, but it happens.” (RMNH provider)*

One provider suggested that a slap could be seen as a “lovely” slap, because it was done with the intention to save the baby’s life. Several providers echoed statements by female clients, suggesting that the sight of a healthy baby will make a woman forget the abuse, or that a provider will apologize or explain such behavior after the birth. At least one provider noted the responsibility of colleagues to address the abuse perpetrated by their peers, and emphasized that rather than “punish” a woman, providers should explain *“what is going to be done to her and how”.*

Providers suggested that maternity services are more likely to include high stress situations that can lead to poor interactions between women and providers, compared to other health services. One provider noted that although midwives sometimes “punish” women, *“a woman in labor can stress you more than another woman”.* Providers highlighted several factors that may contribute to disrespect and abuse, such as exhaustion and stress (due to the limited number of providers compared to clients), poor stress management, personality, and the fear of potential consequences (such as imprisonment) for providers if negative birth outcomes (e.g. death of mother or child) are believed to be due to their negligence:

*“It depends on the personality of the health provider. There are some who get stressed...So, one is stressed because the baby can die, and the stress of what will occur after the baby’s death and the imprisonment, so because of all that, you find yourself having beaten her already without even being aware of it.” (RMNH provider)*

Several providers suggested that providers have less tolerance for misbehavior by multiparous women – especially those in their third or fourth pregnancy – whom they expect to know better. However, disrespectful interactions were also described for first-time mothers:

*“It is not often, but it happens. I remember when I started working, there is someone who told a woman – you see when it is the woman’s first time to deliver, when you tell her to open up the legs, she tends to close them. So, she/he told her, ‘Why didn’t you close your legs when he was impregnating you?’ I am sure that woman didn’t forget that.” (RMNH provider)*

#### Men’s accompaniment during labor and childbirth

A number of female and male clients expressed support for men accompanying their partners to labor and/or childbirth. One man suggested that a man will accompany his wife to, “*know what is going on … and because you love her*”. Overall, female and male clients reported that women were treated with greater respect by providers when accompanied by their male partner. One female client noted that a woman is received differently – better – by health providers when her partner accompanies her. One male client suggested that a man accompanies his wife in order “*to make her respected*”. However, several female and male clients reported that men are not always allowed to be present during labor or birth, which one female client said can cause anxiety. Several clients suggested that men’s ability to be present depends on the attitudes of the providers on duty.

Providers were generally supportive of men’s accompaniment during labor and childbirth and acknowledged men as a source of affection, comfort, encouragement, and support for women. The providers agreed that a woman is allowed to have a birth companion present. One provider stated that a woman’s husband has priority, but emphasized the need for a woman’s consent before allowing her partner into the delivery room. The provider also acknowledged that some women do not want their partner present for the birth. However, several providers acknowledged that men are not always allowed to be present, citing the limited number of private rooms in some facilities (where several women are together in one room) as a barrier.

Several providers suggested that when men are present, they are not always capable to handle the experience. They shared how some men are uncomfortable with their partners being physically examined by a provider or become distressed when witnessing a partner’s pain. A few providers said they have had to ask a man to leave the delivery room, but one provider suggested that you cannot ask a man to leave because it implies that he was not welcome in the first place. Others noted that it was usually men themselves who asked to leave. Providers also noted that men’s lack of experience with childbirth could lead to misunderstandings or accusations of poor-quality care, as described in this example from one provider:

*“There was a man who decided to go out and called my supervisor. He told him, ‘I don’t understand what the health provider is doing.’ So, my supervisor came to help me, but he found that I am done with helping her to deliver. He told me, ‘There is a man who told me to tell you that he can see that his wife is about to die.’ Then I told him that there was no problem, that the woman was screaming, but that it was normal. She didn’t have any other problem, it is just that the baby was right at the exit.” (RMNH provider)*

Several health providers suggested that women whose partners were present at labor or birth were less likely to receive disrespectful or abusive care. Providers said they sometimes request a man’s help to calm his partner, particularly if she is perceived to be “misbehaving”. One provider suggested that, “*allowing the husband to be present in the delivery room, sometimes it is not because of reasons of comfort, sometimes it is because in their mind they are like, ‘please help us with your wife*.’” However, some providers also suggested that in such a situation, a woman’s partner may also be a source of abuse in the delivery room:

“*Even if they said that when a woman is accompanied she is not beaten, sometimes her husband also beats her! Her husband can be like, ‘You stupid, do you want us to lose everything in vain*?’” *(RMNH provider)*

## Discussion

The results of this study indicate that on the whole, the health providers we interviewed understand what high quality, dignified and respectful RMNH care looks like and desire to provide it to their clients. However, these intentions are sometimes undermined by providers’ gendered attitudes and by asymmetries in power between providers and their female clients. In addition, some forms of mistreatment (e.g. slapping) during childbirth appeared to result from providers’ sense of urgency for the safety of the baby and a lack of knowledge of alternative ways to support a woman and facilitate a safe delivery. As a result, women sometimes received low quality RMNH services, or disrespectful or abusive care.

The findings indicate that inequitable gender norms and the gendered attitudes of providers, as well as power dynamics between clients and providers, contribute to women’s experiences of poor quality, disrespectful RMNH care, including disrespect and abuse during childbirth. Gender norms influenced providers’ expectations for — and perceptions of— appropriate behavior when dealing with female and male clients. For example, female clients were expected to be quiet and submissive, as has also been noted in studies from other settings [16, 28]. The qualitative findings suggest women who did not meet these gendered expectations for being quiet and submissive during labor and childbirth – such as those who closed their legs or did not push at the appropriate times – were described as “misbehaving”.

Providers were described as using verbal or physical abuse – such as slapping women – to correct or “punish” women’s behavior, particularly when it was perceived to threaten the baby’s life. Our survey results underscore this finding, with up to a third of providers showing acceptance of verbal or physical abuse during childbirth. The qualitative findings are similar to research from Nigeria and Kenya, where shouting or slapping was reported as justifiable to address women’s perceived disobedience [29, 30]. Similar to other studies, we found that disrespectful and abusive care was sometimes normalized and women were expected to forget or move on from their poor birth experiences [15, 31]. Jewkes and Penn-Kekana assert that disrespect and abuse during childbirth, like intimate partner violence, stem from structural gender inequality, with providers’ behavior influenced by gender norms that devalue women’s position in society, and by their perceived power over clients [31]. They note that providers may use physical violence to maintain control over women and to punish their perceived disobedience.

Other studies have suggested that disrespect and abuse is less likely to occur if a woman has a labor companion to advocate for them [32–34]. Importantly, nearly all of the providers surveyed in this study supported a woman’s right to have a birth companion. In addition, some providers in this study suggested that women who were accompanied by a male partner were less likely to experience mistreatment or disrespectful care from providers during labor and childbirth. This finding echoes reports from women and men in two other studies in Rwanda [35, 36]. In addition, some women in this study reported being treated with greater respect or preference by providers when they were accompanied by a male partner at both ANC and maternity services. These findings have direct implications for the care received by single or unaccompanied women, who may receive delayed or lower-quality care if women attending services with a male partner are prioritized or treated with greater respect by providers.

Many of the health providers in this study were supportive of men’s participation in maternal health services, which likely reflects the increased promotion of men’s engagement within Rwanda’s RMNH policies. However, as men’s engagement increases, health providers and facilities need to be equipped to promote men’s participation and companionship in ways that respect women’s agency and autonomy over their bodies, their health care decisions and preferences for men’s engagement [37–39]. Moreover, strategies to engage men must not penalize or deprioritize women that attend maternal health services without a partner (e.g. women whom are single, widowed, in abusive relationships or whose partners are unavailable) or deny them care [5, 39–41]. Importantly, at least one provider in this study emphasized the importance of gaining a woman’s consent for her partner to be present at childbirth. All providers need to be trained to seek women’s consent for men’s birth companionship and to recognize that women may have diverse preferences – such as not wanting a male partner present at all, or wanting him present during labor, but not at birth [39, 42]. However, this study also highlighted potential risks of men’s participation in maternal health services, such as limiting women’s openness or ability to ask sensitive questions, or even perpetrating verbal or physical abuse against their partners during childbirth.

Overall, the findings highlight the need to challenge health providers’ inequitable gender attitudes and to support them in recognizing imbalanced gender and power dynamics between themselves and their clients, and between female clients and their partners, and how these dynamics can impact the provision and utilization of care [17]. Trainings (including pre- and in-service) that create spaces for health providers to critically reflect on their beliefs and values, question inequitable gender norms, discuss how gender norms and roles can influence provider-client interactions, and to learn and practice more equitable behaviors, can help improve the quality of RMNH care. Such trainings, alongside policy changes and investments in human resources, are a crucial part of transforming inequitable gender norms within the health system. Providers also need to be equipped through capacity building on male engagement to ensure that they apply rights-based approaches to engaging men, and that when men are present, women are able to voice their concerns, and these are addressed and heard [17]. These efforts can be made alongside community interventions, including those promoting men’s engagement in RMNH, that are designed to transform inequitable gender norms and power dynamics and increase women’s agency and autonomy over their bodies and their own health care.

### Limitations

Limitations to this mixed method study include the small sample size for both the qualitative and quantitative studies, the limited number of purposively selected districts, and the limited number of facilities selected for inclusion, which were due to financial and human resource constraints. For the survey, the internal consistency of the scales, as measured by Cronbach’s alpha, while acceptable, is on the low end. Given that Rwanda is a small country and there have been several campaigns and capacity building interventions on gender and QOC issues, it is possible that social desirability bias could have impacted providers’ responses to both the surveys and FGD questions. Further, the qualitative study relied on reported perceptions and self-reported primary individuals’ experiences and observations, rather than direct observation. The selection of eligible clients for the FGDs relied on lists provided by community health workers, which may have introduced an element of selection bias into client recruitment. In addition, the selection criteria for clients meant that the recall period for experiences relating to labor and childbirth was up to 2 years, a significant amount of time that could have affected accuracy of recall. Nevertheless, the similar themes echoed across the qualitative and quantitative data, as well as the diversity of experiences described, provide important insights about gender and quality of care. Finally, it must be noted that given the limited sample size and study locations, the findings described here cannot be considered representative of all health providers, or of all clients’ experiences of RMNH services, or the quality of RMNH care at health facilities in these districts or Rwanda more broadly.

## Conclusion

The findings from this study suggest that inequitable gender norms and attitudes of RMNH providers affect the QOC that women receive, potentially undermining their health and wellbeing and that of their newborn. This threatens progress to reduce maternal and newborn morbidity and mortality [43]. The qualitative results illustrate how gender norms and attitudes – which confer men with greater status than women in society and devalue women’s lives and decisions – influence the provision of care and interactions between providers and clients. The survey results, which found a relationship between health providers’ gender attitudes and their attitudes towards quality, respectful RMNH care – with more equitable attitudes associated with greater support for respectful, quality RMNH care, complement these findings. The study raises concerns for the QOC women receive, particularly for single women or women attending services alone, who may be more likely to experience disrespectful or abusive care. Future research could further explore these findings, including through direct observation of service provision, to inform both RMNH QOC and male engagement programming and policy.

## Supplementary Information

**Additional file 1.** Health provider survey (English).

## Data Availability

Data is available by request from the corresponding author.

## Abbreviations

ANC: Ante-natal care
DHS: Demographic and health survey
FGD: Focus group discussion
FP: Family planning
IMAGES: International men and gender equality survey
JHSPH: Johns Hopkins school of public health
MCSP: Maternal and child survival program
MOH: Ministry of health
OLS: Ordinary least squares
QOC: Quality of care
RBC: Rwanda biomedical center
RMNH: Reproductive, maternal, and newborn health
WHO: World health Organization
