# Supplementary material for: The relationship between inequitable gender norms and provider attitudes and quality of care in maternal health services in Rwanda: a mixed methods study
Source: BMC Pregnancy Childbirth. 2021 Feb 22;21:156. doi: 10.1186/s12884-021-03592-0 (PMC7903699; doi:10.1186/s12884-021-03592-0)
Supplement: Supplementary file 1 — Additional file 1. Health provider survey (English). [file 12884_2021_3592_MOESM1_ESM.pdf]

Study Title: Understanding gender and quality of care for RMNH services in Rwanda: a mixed methods study in three districts

**Thank you for taking the time to complete this survey. Please mark your responses visibly inside the boxes.**<br><br>**By completing this survey you indicate your consent to participate in this study, and have completed a consent form provided to you along with this survey.**<br><br>**After completing the survey, place it in the envelope provided and seal the envelope. The survey will be collected by a representative of Jhpiego/MCSP Rwanda.**<br><br>**Please make sure to place the completed survey in a separate envelope from the signed consent form.**		
This section to be completed by the research team.<br><br>Survey Code ☐☐ ☐☐ ☐☐<br><br>Date ☐☐ ☐☐ ☐☐		
1	How old are you?	☐ ☐ years
2	What is your gender?	☐ Female ☐ Male
3	What is your occupation?	☐ Midwife (A1) ☐ Doctor (generalist)<br>☐ Nurse (A1) ☐ Doctor (specialist)<br>☐ Nurse (A2) ☐ Other<br>☐ Nurse (A3)
4	Which of the following types of health care services are you involved in? [MARK ALL THAT APPLY.]	☐ Family planning ☐ Postnatal care<br>☐ Antenatal care ☐ Other service<br>☐ Labor/delivery
**Mark whether you strongly agree, agree, disagree, or strongly disagree with each of the statements below.**		
5	A woman's most important role is to take care of her home and cook for her family.	☐ Strongly agree ☐ Disagree<br>☐ Agree ☐ Strongly disagree

6	It is natural and right that men have more power than women.	☐ Strongly agree<br>☐ Agree	☐ Disagree<br>☐ Strongly disagree
7	It is a woman's responsibility to avoid getting pregnant.	☐ Strongly agree<br>☐ Agree	☐ Disagree<br>☐ Strongly disagree
8	A man should have the final word about decisions in his home.	☐ Strongly agree<br>☐ Agree	☐ Disagree<br>☐ Strongly disagree
9	There are times when a woman deserves to be beaten.	☐ Strongly agree<br>☐ Agree	☐ Disagree<br>☐ Strongly disagree
10	Men are better at making decisions than women are.	☐ Strongly agree<br>☐ Agree	☐ Disagree<br>☐ Strongly disagree
11	Women should remain virgins until they get married.	☐ Strongly agree<br>☐ Agree	☐ Disagree<br>☐ Strongly disagree
12	Men should be as involved in caring for their children as women are.	☐ Strongly agree<br>☐ Agree	☐ Disagree<br>☐ Strongly disagree
13	A woman should not use a family planning method unless her partner agrees.	☐ Strongly agree<br>☐ Agree	☐ Disagree<br>☐ Strongly disagree
14	A woman who uses contraceptives without telling her husband deserves to be beaten.	☐ Strongly agree<br>☐ Agree	☐ Disagree<br>☐ Strongly disagree
15	A woman who has not undergone *gukuna imishimo* does not deserve respect from her husband.	☐ Strongly agree<br>☐ Agree	☐ Disagree<br>☐ Strongly disagree
16	Adolescents seeking contraceptives should be advised to abstain from sex.	☐ Strongly agree<br>☐ Agree	☐ Disagree<br>☐ Strongly disagree
17	Male health providers have more power and are more respected than female health care providers.	☐ Strongly agree<br>☐ Agree	☐ Disagree<br>☐ Strongly disagree

18	A girl who gets pregnant before marriage deserves to be shunned, sent away, or otherwise punished.	☐ Strongly agree<br>☐ Agree	☐ Disagree<br>☐ Strongly disagree
19	A good client never questions a health provider's decisions, even if s/he disagrees with them.	☐ Strongly agree<br>☐ Agree	☐ Disagree<br>☐ Strongly disagree
20	A woman should be able to use contraceptives, even if her partner disagrees.	☐ Strongly agree<br>☐ Agree	☐ Disagree<br>☐ Strongly disagree
21	Health providers should show sympathy and care to women during childbirth.	☐ Strongly agree<br>☐ Agree	☐ Disagree<br>☐ Strongly disagree
22	It is better for a health provider to be decisive than to explain everything to a client/patient.	☐ Strongly agree<br>☐ Agree	☐ Disagree<br>☐ Strongly disagree
23	It's easier to work with women when they come to ANC with their partners.	☐ Strongly agree<br>☐ Agree	☐ Disagree<br>☐ Strongly disagree
24	A woman should be allowed to have a companion present at delivery, if she chooses, and without any further explanation.	☐ Strongly agree<br>☐ Agree	☐ Disagree<br>☐ Strongly disagree
25	It is important for health care providers assert their power over their clients in order to be respected.	☐ Strongly agree<br>☐ Agree	☐ Disagree<br>☐ Strongly disagree
26	When health providers listen to their clients/patients' questions and concerns it results in better service provision.	☐ Strongly agree<br>☐ Agree	☐ Disagree<br>☐ Strongly disagree
27	Sometimes you have to yell or shout at a woman while she is giving birth to get her to push.	☐ Strongly agree<br>☐ Agree	☐ Disagree<br>☐ Strongly disagree
28	Men should not be present when women are giving birth.	☐ Strongly agree<br>☐ Agree	☐ Disagree<br>☐ Strongly disagree
29	Sometimes women need a little push or slap to motivate them during childbirth.	☐ Strongly agree<br>☐ Agree	☐ Disagree<br>☐ Strongly disagree

30	A good woman keeps quiet during delivery, even when she is in pain.	☐ Strongly agree<br>☐ Agree	☐ Disagree<br>☐ Strongly disagree
31	A woman should be able to get help from a skilled birth attendant when she needs it, even without her husband's approval.	☐ Strongly agree<br>☐ Agree	☐ Disagree<br>☐ Strongly disagree
32	If a woman is unable to pay for the services she has received, she should be detained in the facility until payment is made.	☐ Strongly agree<br>☐ Agree	☐ Disagree<br>☐ Strongly disagree
